# The Role of Crosslinking Agents in the Development of Collagen–Hydroxyapatite Composite Materials for Bone Tissue Engineering

**DOI:** 10.3390/ma18050998

**Published:** 2025-02-24

**Authors:** Alina Florentina Vladu, Madalina Georgiana Albu Kaya, Roxana Doina Truşcă, Ludmila Motelica, Vasile-Adrian Surdu, Ovidiu Cristian Oprea, Rodica Roxana Constantinescu, Bogdan Cazan, Denisa Ficai, Ecaterina Andronescu, Anton Ficai

**Affiliations:** 1 The National Research and Development Institute for Textiles and Leather, Lucretiu Patrascanu, 030508 Bucharest, Romania; alina_vladu_1995@yahoo.com (A.F.V.); bogdan.cazan@incdtp.ro (B.C.); 2 Science and Engineering of Oxide Materials and Nanomaterials, Faculty of Chemical Engineering and Biotechnologies, National University of Science and Technology POLITEHNICA Bucharest, Gh. Polizu 1-7, 011061 Bucharest, Romania; truscaroxana@yahoo.com (R.D.T.); motelica_ludmila@yahoo.com (L.M.); adrian.surdu@upb.ro (V.-A.S.); ovidiu.oprea@upb.ro (O.C.O.); ecaterina.andronescu@upb.ro (E.A.); anton.ficai@upb.ro (A.F.); 3 National Center for Micro and Nanomaterials, National University of Science and Technology POLITEHNICA Bucharest, Splaiul Independentei 313, 060042 Bucharest, Romania; denisa.ficai@upb.ro; 4 National Center for Scientific Research for Food Safety, National University of Science and Technology POLITEHNICA Bucharest, Splaiul Independentei 313, 060042 Bucharest, Romania; 5 Division of Leather and Footwear Research Institute, The National Research and Development Institute for Textiles and Leather, 93 Ion Minulescu Str., 031215 Bucharest, Romania; rodica.roxana@yahoo.com; 6 Academy of Romanian Scientists, Ilfov Street 3, 050044 Bucharest, Romania; 7 Research Center for Advanced Materials, Products and Processes, National University of Science and Technology POLITEHNICA Bucharest, 060042 Bucharest, Romania; 8 Department of Inorganic Chemistry, Physical Chemistry and Electrochemistry, Faculty of Chemical Engineering and Biotechnologies, National University of Science and Technology POLITEHNICA Bucharest, Gh. Polizu 1-7, 011061 Bucharest, Romania

**Keywords:** composite materials, bone tissue engineering, freeze-drying, in situ hydroxyapatite

## Abstract

The lack of bone grafts represents a major issue in the orthopedic field, reconstructive surgery, and dentistry. There are several bone conditions that often demand the use of grafts, such as fractures, infections, and bone cancer. The number of bone cancer cases increased in the past few decades and along with it, the need for bone grafting materials. To avoid the use of autografts and allografts there has been an increased interest towards synthetic grafts. This research aims to develop some collagen/hydroxyapatite (Coll/HAp) scaffolds cross-linked with three different agents that could be used in bone tissue engineering (BTE). These scaffolds were obtained with a freeze-drying method after the in situ formation of hydroxyapatite inside the collagen matrix. They were structurally and morphologically characterized and evaluated in terms of antimicrobial activity on *E. coli* and *S. aureus* bacterial strains. The results revealed that the scaffolds have porous structures with interconnected pores of suitable dimensions and well-distributed inorganic phases. Coll/HAp samples showed great antibacterial activity even without the use of typically used antibacterial agents. These findings allow us to conclude that these scaffolds are promising candidates for use in BTE and bone cancer treatment after the incorporation of specific antitumoral drugs.

## 1. Introduction

Considering the increasing number of orthopedic surgeries that are performed globally, one of the most important issues associated with these procedures is the reconstruction of bone defects that are the result of trauma, congenital deformity, and tumor removal [1]. The worldwide bone grafts and substitutes market size was rated at 3.16 billion USD in 2024 and is expected to grow by 6.6% from 2025 to 2030. There is a growing demand for the use of synthetic substitutes, and an increasing amount of product approval by regulatory authorities is expected to lead to market growth in the following period [2]. An ideal bone substitute should be osteoinductive, osteoconductive, and allow osteogenesis. It should also be easy to harvest and available in the needed quantities, as well as possess a minimal risk for infection transmission and be inexpensive [3].

**Figure 1:**
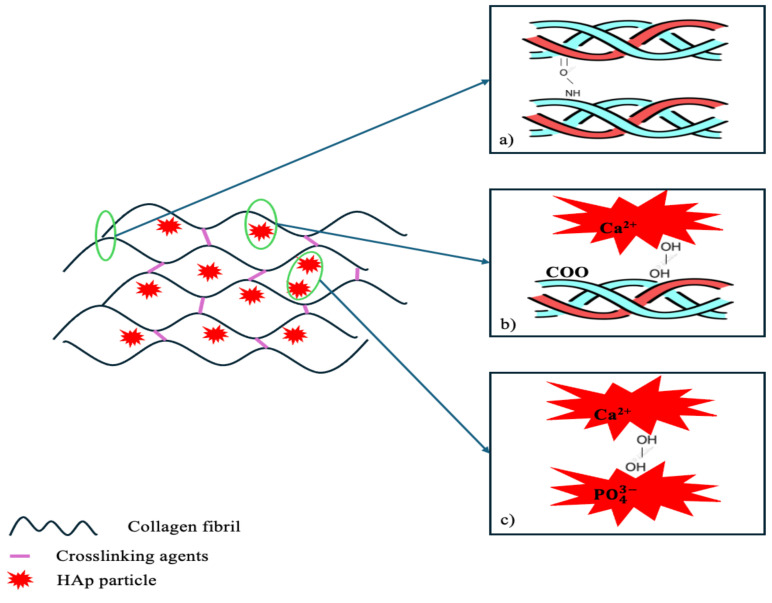
The mechanism of crosslinking and collagen–hydroxyapatite interactions: (**a**) intermolecular collagen interactions, (**b**) collagen-hydroxyapatite interactions and (**c**) hydroxyapatite-hydroxyapatite interactions.

However, the demand for bone grafts significantly exceeds the available supply, particularly autografts, which remain the gold standard despite limitations such as scarce supply and donor site morbidity [4,5,6]. Bone tissue is made up of both organic and inorganic components and features a highly organized structure [7]. As a result, there has been growing interest in developing synthetic biomaterials that can either supplement or replace natural bone grafts. Among these, collagen and hydroxyapatite (HAp)-based materials have garnered considerable attention due to their biocompatibility and osteoconductive properties, which are essential for promoting bone regeneration [8]. Collagen, particularly type I collagen, is a primary element of the extracellular matrix (ECM) and is widely found in skin, bones, tendons, and organs. Its triple-helix structure and the presence of the RGD sequence make it ideal for supporting cellular functions [9,10]. Hydroxyapatite, a naturally occurring calcium phosphate mineral, is widely used in bone grafts due to its chemical similarity to bone mineral and its strong osteoconductive properties [11]. It forms on specific sites of collagen fibers deposited by osteoblasts, the cells responsible for bone formation. Research indicates that HAp-based bone graft substitutes perform comparably to bone allografts in terms of long-term clinical outcomes, highlighting their effectiveness as a biomaterial for bone replacement [12,13]. There are several well-known methods for obtaining composite biomaterials including freeze-drying, biomimetic mineralization, electrospinning, and 3D printing [14]. In tissue engineering, HAp-based scaffolds face challenges due to the low mechanical strength and high solubility of the polymers used, which can lead to rapid degradation in cell culture environments. To address these issues, various crosslinking methods have been developed to enhance the mechanical properties of these composites. Crosslinking increases the material’s Young’s modulus and reduces its swelling, making it crucial for improving both the durability and stability of HAp-based scaffolds [14,15,16]. Collagen crosslinking is a process where collagen molecules or fibers are chemically or physically bonded to enhance their structural integrity, stability, and mechanical properties. This is achieved using crosslinking agents that form bridges between collagen molecules, fibrils, or fibers (Figure 1). These bridges can occur via covalent or non-covalent interactions, depending on the type of crosslinking agent and reaction conditions [17]. In COLL/HA composites, there are also several interactions between the components. The main interactions include hydrogen bonding and electrostatic interactions. Hydrogen bonds form within and between collagen molecules, fibrils, fibers, and hydroxyapatite. Electrostatic interactions occur primarily between calcium ions in hydroxyapatite and carboxylate groups in collagen, as well as among hydroxyapatite grains and charged groups within collagen. These interactions contribute to the structural and functional integration of the composite [18].

Glutaraldehyde (GTA) is a widely used bifunctional crosslinking agent for stabilizing fibrous proteins like collagen. It forms imino bonds with amino groups in lysine or hydroxylysine residues, enhancing protein stability. GTA is favored in biomedical applications due to its affordability, availability, high reactivity, and strong stabilizing properties [19]. However, its use is limited by local cytotoxicity, which can trigger unwanted immune responses. This toxicity is concentration-dependent, with up to 8% GTA being considered safe [20]. Washing scaffolds with a glycine solution can further reduce toxicity by removing unbound aldehyde groups. GTA has been utilized in bone tissue engineering, such as in gelatin–HAp electrospun fiber scaffolds and Alg-HAp hydrogels, often combined with freeze-drying or electrospinning methods. Despite its benefits, careful management of GTA’s concentration and residuals is necessary to ensure biocompatibility [13]. Wu et al. evaluated a biogenic collagen membrane enhanced through GTA–alendronate cross-linking and zinc-doped nanohydroxyapatite doping. The modified membrane demonstrated extended biodegradation, satisfactory biocompatibility, and favorable physicochemical properties, making it well-suited for clinical applications [21]. Rahman et al. fabricated some hydroxyapatite/collagen/chitosan biomaterials crosslinked with glutaraldehyde and IR radiation using a thermally induced phase separation technique for the restoration of defected mandible bone. Composites crosslinked with IR radiation or glutaraldehyde showed improved hydrophilicity and biodegradability compared to other methods. Additionally, all materials tested demonstrated biocompatibility and no cytotoxicity [22,23].

Genipin is a naturally occurring cross-linking agent widely recognized for its excellent biocompatibility, biodegradability, and the stability of its cross-linked products. It specifically reacts with primary amine groups, making it suitable for use with biomaterials like chitosan and proteins. The cross-linking process with genipin is considered mild and environmentally friendly. Compared to other cross-linkers, genipin is especially attractive due to its significantly lower cytotoxicity, being about 10,000 times less toxic than glutaraldehyde [24,25]. Moreover, genipin is a powerful colorant and a great antioxidant, antimicrobial, and anticancer compound [26]. Ma et al. prepared collagen-based hydrogels cross-linked with genipin, which demonstrated enhanced mechanical strength, reduced swelling ratios, and increased gel content compared to pure collagen hydrogels. Additionally, these cross-linked hydrogels exhibited tunable degradation properties when exposed to collagenase [27]. Scialla et al. evaluated genipin-crosslinked collagen–hydroxyapatite (Col-HA) scaffolds, which promoted chondrogenesis, showing isotropic and homogeneous pore distribution. The presence of genipin contributed to a more uniform chondral-like matrix deposition by human mesenchymal stem cells (hMSCs) [28]. Regarding genipin’s antimicrobial activity, Huertas-Bello et al. studied the antibacterial activity of genipin against *Pseudomonas aeruginosa*, *Candida albicans,* and *Staphylococcus aureus* on an ex vivo porcine corneal model. According to the results, genipin successfully inhibits the growth of these three strains, emphasizing its potential to treat severe microbial keratitis [29].

Tannic acid (TA) is a natural polyphenol found in various plants, appearing as a colorless to yellowish solid with a tangy flavor. It can be extracted from sources such as oak bark, chestnut, hemlock, mangrove, certain sumac leaves, and plant galls [30]. It has been intensively studied due to its unique antiviral and antibacterial properties. The antibacterial properties of tannins are linked to their capacity to penetrate the bacterial cell wall and reach the internal membrane, interfering with their biochemical processes, and leading to their death [31]. Tannic acid (TA), due to its unique polyhydric phenol structure, can act as both a physical and chemical crosslinker, enabling the formation of versatile polymeric networks. In physical or supramolecular crosslinking, TA interacts with macromolecules at multiple binding sites through hydrogen bonding, ionic bonding, and π–π/cation–π interactions, allowing it to effectively complex or crosslink various materials. Tannic acid (TA) not only enables physical crosslinking for supramolecular materials but also chemically reacts with other functional molecules to create covalent polymeric networks. This versatility in TA-based polyphenol chemistry allows the development of functional polymers with applications in adhesive hydrogels, drug delivery nanocarriers, thermosets, and tissue engineering, among other fields [32,33]. Kaczmarek et al. obtained some scaffolds composed of collagen, chitosan, and hyaluronic acid supplemented with nano-hydroxyapatite and tannic acid using a freeze-drying technique, which were implanted into the rabbit bone. The results indicated that incorporating tannic acid enhances the material’s compatibility with SaOS cells. In vivo studies on rabbit bone tissue demonstrated that the biomaterials exhibited good compatibility with surrounding tissues. Observations included calcification sites, angiogenesis, and macrophage response to the implanted materials. The presence of blood vessels and bone matrix formation further confirmed that the bone tissue regeneration process was successfully initiated [34]. Dong et al. studied the antimicrobial activity and the mechanism of action of tannic acid against a pathogenic bacteria, *Staphylococcus aureus*, emphasizing its ability to inhibit biofilm formation. The results showed great antimicrobial and anti-biofilm effects, suggesting its ability to combat *S. aureus* infections [35]. This paper is focused on the evaluation of two series of composite materials based on collagen and hydroxyapatite with Coll/HAp ratios of 3:1 and 1:1, designed to be used in bone tissue engineering. Each series contains an uncrosslinked sample and three samples crosslinked with three different agents (glutaraldehyde, tannic acid, and genipin). The selection of two ratios with high collagen content and three crosslinking agents was made to increase the biodegradability rates. As the organic part is broken down, the inorganic phase remains, which will be used by the organism to form new bone.

## 2. Materials and Methods

The synthesis of collagen–hydroxyapatite (Coll/HAp)-based composite materials was achieved using type I collagen (Coll) gel, which was obtained from bovine skin using a method developed by the Collagen Research Department at INCDTP—Leather and Footwear Research Institute [34], with a collagen concentration of 1.67% (*w*/*v*). For the in situ synthesis of hydroxyapatite, calcium hydroxide—Ca(OH)_2_ of ≥96% purity from Fluka (Buchs, Switzerland) and di-ammonium hydrogen phosphate—(NH_4_)_2_HPO_4_ of ≥97% purity from Roth (Karlsruhe, Germany) were used. Sodium hydroxide—NaOH of 99.45% purity was purchased from Lach-Ner (Neratowitz, Czech Republic). The crosslinking agents, tannic acid, glutaric dialdehyde solution of 25% *w*/*v*, and genipin of ≥98% purity, were obtained from Sigma Aldrich (St. Louis, MA, USA). All reagents were used without further purification.

### 2.1. Obtaining the Freeze-Dried Coll/HAp Composite Materials

Coll/HAp composite materials of different ratios (3:1 and 1:1) were synthesized using a freeze-drying technique of samples with the composition according to Table 1.

**Figure 2:**
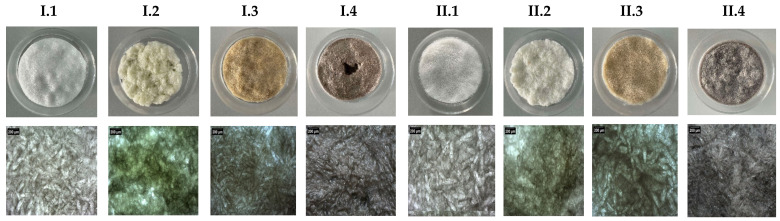
Macroscopic and microscopic (×2) view of the obtained composite materials.

A Ca(OH)_2_ suspension and (NH_4_)_2_HPO_4_ solution were prepared, and the collagen gel was diluted to a concentration of 1% (*w*/*v*). The precursors were mixed in order to obtain a molar ratio of Ca/P of 1.67. First, the Ca(OH)_2_ suspension was added under constant stirring in the collagen gel. For the in situ synthesis of hydroxyapatite, the (NH_4_)_2_HPO_4_ solution was added dropwise over the gel and stirred for about 1 h, while the pH was kept at 9.5 by using NaOH. The Coll/HAp gel was washed by introducing it into a dialysis membrane and sinking it in distilled water for 24 h. The gel was then crosslinked with three different crosslinking agents (glutaraldehyde, tannic acid, and genipin) and poured into glass Petri dishes with a 5.2 cm diameter and 1 cm height. A Delta LSC 2-24 lyophilizer (Martin Christ, Osterode am Harz, Germany) was used to dry the obtained samples. Freeze-drying was necessary to obtain spongy forms (matrices/sponges). Samples were placed on the lyophilizer shelves pre-cooled to −40 °C for one and a half hours. Freeze-drying was carried out for 48 h, in 11 stages. Figure 2 shows the obtained composite structures. The microscopic images captured with a Nikon SMZ745T stereomicroscope (Tokyo, Japan) emphasize the porous structure similar to a sponge. By carefully viewing the images, we can appreciate that the pore sizes are in the micrometric range, although more detailed information was obtained by association with other, more powerful, characterization techniques.

### 2.2. Characterization Techniques

#### 2.2.1. Water Absorption of the Composite Materials

The water absorption capacity of the composite materials was measured by weighing a small piece of sample (0.0072–0.0141 g) before and after immersion in 3 mL of water at a temperature of 25 °C. The composites were reweighed at specific intervals (1, 2, 4 h; 1, 2, 3 days), and their water retention capacity was calculated using Equation (1):

(1) $$\mathrm{Weight uptake}\;(\%)=\frac{W_{t}-W_{d}}{W_{d}}100$$

where Wt is the absorbed water weight in the composites at time t, and Wd is the weight of the dry composites. The data are presented as the mean ± standard deviation (SD) derived from three separate experiments [36].

#### 2.2.2. Enzymatic Degradation

The enzymatic degradation of Coll/HAp composites was performed by submerging hydrated sponges in a collagenase solution and tracking their breakdown over time. To assess weight reduction, samples were periodically taken from the solution and weighed at specific time points (1, 2, 3, and 4 days). The weight loss was determined using the following equation (Equation (2)):

(2) $$\mathrm{Weight loss}\;(\%)=\frac{W_{i}-W_{t}}{W_{t}}100$$

where Wi is the initial weight, and Wt is the weight of the sample after time t [37]. The data are presented as the mean ± standard deviation (SD) derived from three separate experiments.

#### 2.2.3. Scanning Electron Microscopy (SEM) and ImageJ Porosity Determination

The samples were analyzed using scanning electron microscopy (SEM) to examine the surface morphology of the composite materials, assess the distribution of hydroxyapatite within the polymer matrix, and evaluate pore size and appearance. The images were captured with a Quanta Inspect F50 (FEI Company, Eindhoven, The Netherlands) SEM equipped with a field emission gun (FEG), providing a resolution of 1.2 nm and an energy-dispersive X-ray spectrometer with an MnK resolution of 133 eV. The porosity of the samples was evaluated using ImageJ Java 1.54g-based processing program.

#### 2.2.4. Fourier Transform Infrared Spectroscopy (FTIR)

Fourier transform infrared (FTIR) spectroscopy was used to investigate the presence and interactions of specific functional groups within the 400–4000 cm^−1^ range, employing a Nicolet iS50 spectrometer (Thermo Fisher Scientific Inc., Waltham, MA, USA). The spatial distribution of the components was further analyzed using FTIR microscopy, utilizing a Nicolet iN10 MX microscope (Thermo Fisher Scientific Inc., Waltham, MA, USA).

#### 2.2.5. X-Ray Diffract Ion (XRD)

X-ray diffraction (XRD) was performed at room temperature to determine the phase composition and crystal structure of the obtained scaffolds. The device that was used was a PANalytical Empyrean diffractometer (Almelo, Netherlands), which used a Ni-filtered Cu-Kα radiation (λ = 1.5418 Å), in θ–2θ mode. The 2θ ranged (20–60°). The step size was 0.02°, and the counting time per step was 255 s.

#### 2.2.6. Thermogravimetric Analysis

Thermal stability was analyzed using STA 449C F3 TG-DSC (thermogravimetry–differential scanning calorimetry) materials from Netzsch (Selb, Germany) in a dynamic air atmosphere (50 mL/min) over a temperature range from 20 to 900 °C. The gases released during the process were transferred through heated lines and continuously analyzed using a Bruker FTIR Tensor 27 spectrometer (Ettlingen, Germany) equipped with an internal thermostatic gas cell.

#### 2.2.7. Mechanical Testing

The compression test used an Instron 34TM-10 (Norwood, Massachusetts, USA) universal testing machine according to the ASTM D695 standard [38]. The test speed was 1 mm/min, and the test’s stopping criterion reached a displacement of 10 mm. The equivalent modulus of elasticity was determined on the linear portion, in the 0–20% area; the initial slope of the curve and the compressive yield limit were determined at 0.2%. The results are presented as the average value ± standard deviation. Two determinations per set were performed.

#### 2.2.8. Antimicrobial Activity

The testing of the samples was carried out on ATCC strains belonging to the ICPI Biotechnology Laboratory, specifically *Escherichia coli* (ATCC 10536) and *Staphylococcus aureus* (ATCC 6538), using a diffusimetric method. A test tube containing Mueller Hinton Broth (MHB) inoculated with *E. coli* and *S. aureus* was incubated for 18 h at 37 °C. Decimal dilutions up to 10^−5^ cfu/mL were made from this culture. A total of 200 μL of the inoculum was plated, followed by the addition of the prepared samples. The plates were incubated for 24 h at 37 °C. The antibacterial activity was then assessed by measuring the diameter of the inhibition zone surrounding the sample.

## 3. Results and Discussion

### 3.1. Water Uptake Evaluation of Coll/HAp Sponges

**Figure 3:**
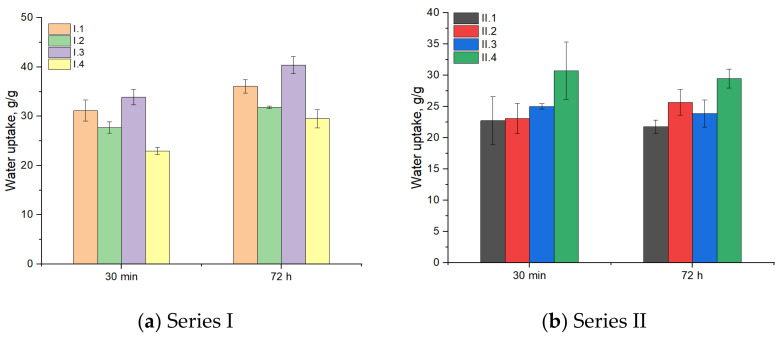
Evaluation of water uptake of composite samples.

Figure 3 presents the diagram corresponding to water absorption, and it can be observed that all samples absorb high amounts of water, between about 23 and 40 g/g.

The majority of the samples retain a considerable amount of water immediately after immersion and a few hours after, and then, the uptake reaches equilibrium. Hydroxyapatite induces morphological changes by reducing the water absorption in samples containing it, making the samples denser. The crosslinking agent doesn’t seem to have a big influence on the water uptake capacity. The water uptake of series I and II at intermediate times is presented in Table A1 and Table A2, which can be found in Appendix A.

### 3.2. Enzymatic Degradation of Coll/HAp Sponges

**Figure 4:**
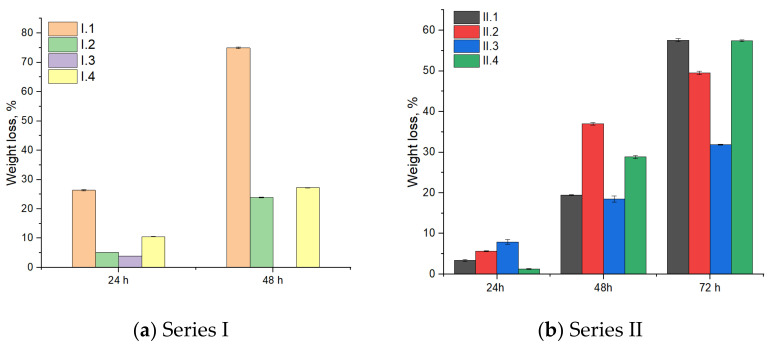
Enzymatic degradation of composite samples.

The degradation of the samples was then evaluated using a collagenase solution, an enzyme that breaks down collagen into amino acids. The weight loss noticed for all samples is shown in Figure 4. An increase in mass reduction is observed over the 96-h period for all eight samples. Complete degradation occurred within 72 h for all samples containing a lower amount of hydroxyapatite, likely due to the simultaneous release of hydroxyapatite and the enzymatic degradation of collagen fibers. Samples containing higher amounts of hydroxyapatite show slower degradation, probably because the collagen matrix is protected by the mineral mass. For a better interpretation of the degradation kinetics, further in vivo studies are needed.

### 3.3. Scanning Electron Microscopy (SEM) and Energy Dispersive X-Ray Spectroscopy (EDX) Characterization

**Figure 5:**
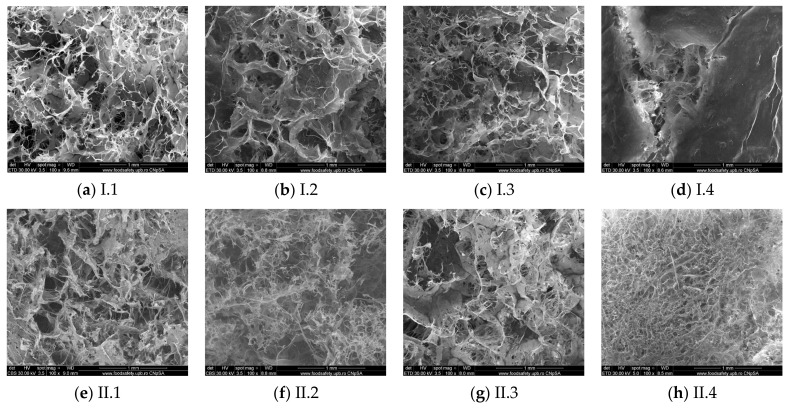
The SEM micrographs obtained for the composite scaffolds (×100): (**a**) I.1, (**b**) I.2, (**c**) I.3, (**d**) I.4, (**e**) II.1, (**f**) II.2, (**g**) II.3, (**h**) II.4.

The obtained samples were further analyzed using scanning electron microscopy to examine the surface morphology of the composite materials, the distribution of hydroxyapatite within the polymer matrix, and to evaluate both the appearance and size of the pores. According to Figure 5, all samples exhibited a spongy structure with pore sizes ranging from 50 to 300 µm, which is optimal for bone scaffold applications. The analysis of these images revealed the fibrillar structure of the collagen matrix. Freeze-drying enhanced the scaffold’s morphology, resulting in an open structure with interconnected pores and a well-distributed pore network. No significant morphological differences were observed between the samples; the only exception was made by composites I.4 and II.4, which appear more compact.

**Figure 6:**
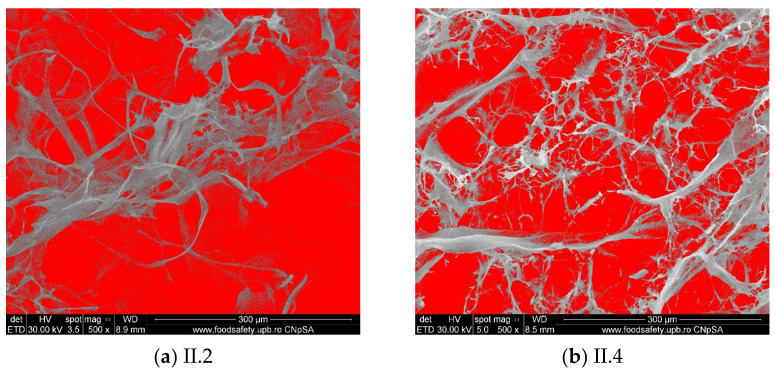
The porosity of composite materials.

The porosity of the composite materials was calculated with the tools provided by the ImageJ program (Figure 6), and the obtained values were placed between 60 and 75%. The calculation was conducted on an area of ~0.27 mm^2^. There were no significant differences regarding the crosslinking agent except for the samples crosslinked with genipin, which appear to be more compact and with smaller pores. All the obtained composite materials have interconnected pores with sizes placed between 50 and 300 μm, which is optimal for osteoblast infiltration and spreading.

**Figure 7:**
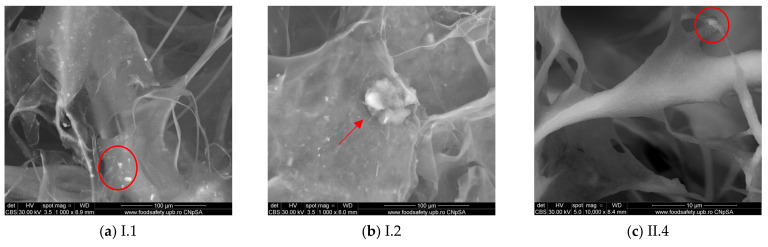
The SEM micrographs obtained for (**a**) I.1 (×1000), (**b**) I.2 (×1000), and (**c**) II.4 (×10,000).

Figure 7 shows the presence of an inorganic phase, the agglomerates of various sizes appear to be homogeneously distributed on the collagen fibers. The agglomerates visible on the scans have dimensions between 1 and 10 μm (Figure 5a). There are also some isolated bigger agglomerates, as shown in Figure 5b, of about 50 μm, as the red arrows show. Collagen fibers presented in sample II.4 appear more homogeneously mineralized than the others, and the hydroxyapatite is evenly covering the fibrils.

By combining scanning electron microscopy with energy-dispersive X-ray analysis, a spectrum detailing the elemental composition of the samples was acquired. This technique identified elements such as Ca, P, Na, Cl, O, and C (Table 2). The presence of calcium, phosphorus, and oxygen can be linked to the presence of hydroxyapatite in the composition, information supported also by the atomic percentage of Ca and P. In the case of sample II.4, the atomic ratio between Ca and P is 1.68, which is close to that of stoichiometric hydroxyapatite. The elemental composition was registered for the area pictured in Figure 7c of 653 μm^2^, and the value of 1.68 is an average that characterizes the entire surface. Additionally, notable amounts of sodium and chlorine were detected, likely originating from NaCl used in the collagen production process, which is non-toxic at these concentrations [39].

### 3.4. FTIR Spectroscopy and Microscopy

**Figure 8:**
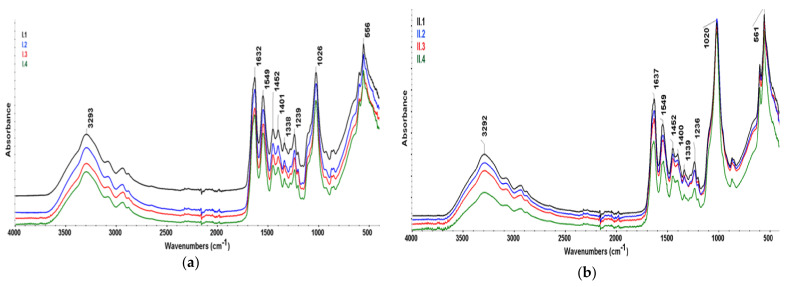
FTIR spectra of (**a**) Col/HAp 3:1 and (**b**) Col/HAp 1:1 composite materials.

By analyzing the FTIR spectra (Figure 8) of the composite structures, the characteristic bands corresponding to the organic and inorganic phases are clearly visible.

The main collagen-specific absorption bands can be easily identified at 3293 cm^−1^ (amide A, N-H stretching), 3082 cm^−1^ (amide B), 2933 cm^−1^ (vCH_2_ symmetric), 2887 cm^−1^ (vCH_2_ asymmetric), 1632 cm^−1^ (vC=O amide I—α helix), 1549 cm^−1^ (amide II), 1452 cm^−1^ (δNH)—pyrrolidine ring, 1401 cm^−1^ (vCOO−), and 1239 cm^−1^ (amide III) and represent N–H bending vibrations coupled with C–H stretching and C–N stretching vibrations [40]. The bands characteristic of hydroxyapatite are also present, although there is a certain overlapping with those of collagen, more precisely in the 3200–3500 cm^−1^ (-OH stretching) and 1400–1632 cm^−1^ region (carbonyl stretching). The peaks that are easily identified and specific to HAp are those belonging to the ${PO}_{4}^{3-}$ group at 1026, 556, and 615 cm^−1^ and associated with symmetric stretching and bending vibrations [41]. The absorption bands of samples for series II are slightly shifted. For samples belonging to series I, a small shoulder at 950 cm^−1^ can be observed, probably attributed to brushite (${HPO}_{4}^{2-})$. The small peak at 872 cm^−1^ belongs to carbonate group ${CO}_{3}^{2-}$. When comparing the obtained spectra with that available in the literature for the hydroxyapatite extracted from natural bone, it can be observed that they present great similarity, the main phosphate band being very sharp and close to 1020 cm^−1^ [42]. Therefore, it can be assessed that the composite materials contain a bone-like apatite phase.

**Figure 9:**
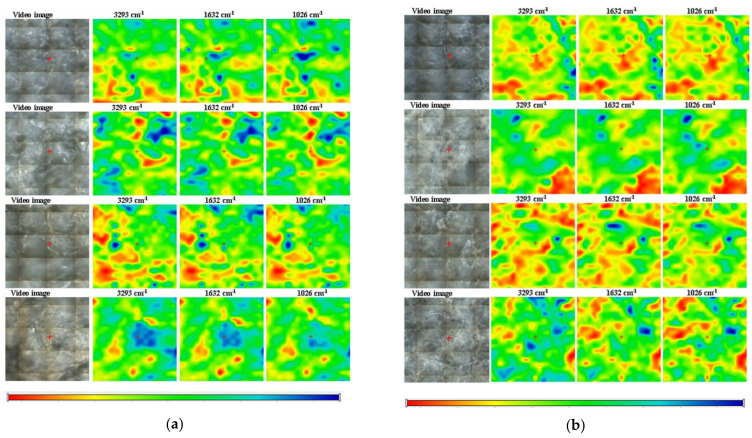
FTIR microscopy maps of (**a**) Coll/HAp 3:1 and (**b**) Coll/HAp 1:1 composite materials; red zones represent areas of maximum absorbance, while blue zones indicate minimal absorbance.

The FTIR microscopy maps for Coll/HAp 3:1 and Coll/HAp 1:1 are presented in Figure 9 at three specific wavenumbers: 3293 cm^−1^, associated with O-H stretching vibration in collagen and hydroxyapatite, 1632 cm^−1^, specific to amid I from collagen, and 1026 cm^−1^, attributed to hydroxyapatite’s ${PO}_{4}^{3-}$ group. All samples showed a high degree of homogeneity at the microscopic level, from the point of view of the distribution of the main components (HA and collagen) but also from the point of view of the humidity distribution, associated with the peak from ~3300 cm^−1^.

### 3.5. X-Ray Diffraction (XRD)

**Figure 10:**
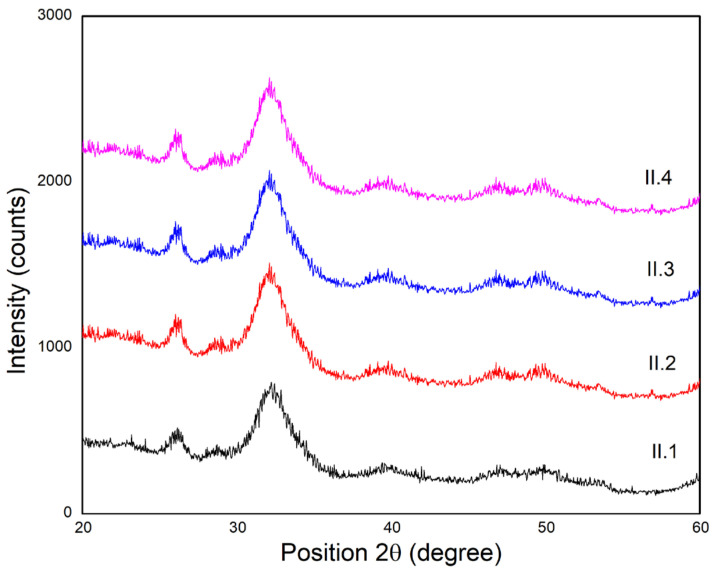
X-ray diffraction patterns for the scaffolds containing Coll/HAp 1:1.

X-ray diffraction patterns were recorded for the confirmation of the in situ synthesis of hydroxyapatite. Figure 10 emphasizes that more amorphous hydroxyapatite was formed inside the collagen matrix. The patterns resemble that of natural bone and show broadened peaks at the positions specific for hydroxyapatite [43]. The broad peaks are also an indication of the nanosized hydroxyapatite formation. The most important and intense peaks are found near the 2θ angles of 26 and 32°, which represent the main characteristic peaks of HAp. Crosslinking agents do not influence the crystallinity of the samples, they are added only after the mineralization process is finalized. No additional crystalline phases are detectable, HAp being the only crystalline phase. The XRD patterns are in agreement with FTIR spectra, confirming the obtained composite materials contain an amorphous bone-like apatite phase, very similar to that found in natural bone, indicating the biomimetic approach [44]. Although the results show a more amorphous apatite, the Ca/P ratio is very close to that of bone, and this corresponds to a Ca/P of ~1.66, specific to hydroxyapatite.

### 3.6. Thermogravimetric Analysis

**Figure 11:**
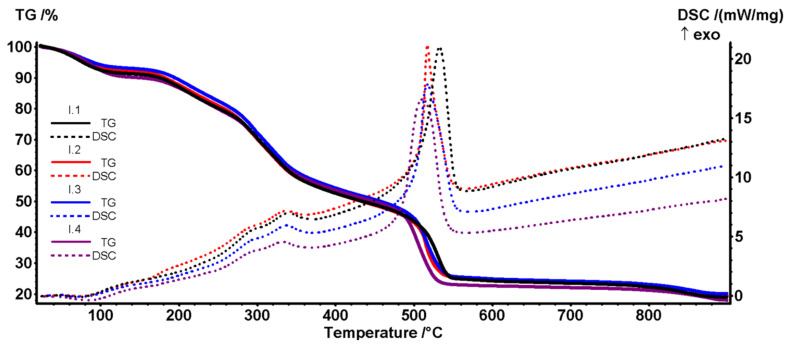
TG-DSC curves for series I (I.1. I.2, I.3 and I.4) containing Coll/HAp 1:3.

**Figure 12:**
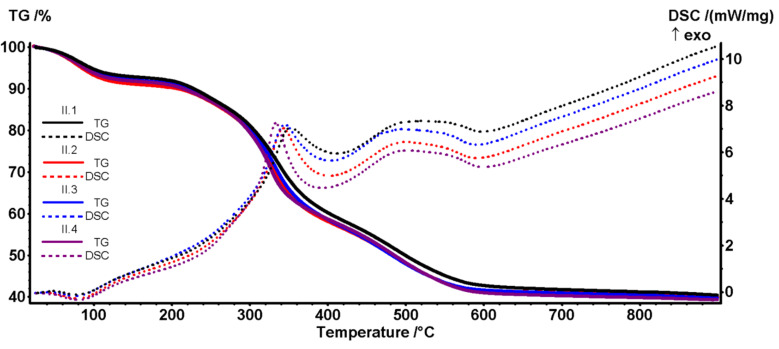
TG-DSC curves for series II (II.1. II.2, II.3, and II.4) containing Coll/HAp 1:1.

The thermal analysis TG and DSC curves for both series I (Coll/HAp 1:3) and II (Coll/HAp 1:1) are displayed in Figure 11 and Figure 12. As can be seen in Table 3, the denaturation of the collagen triple helix takes place at low temperatures, between 20 and 50 °C [45], a function of the collagen origin and crosslinking type [46]. At this temperature, the ordered triple helical structure is lost and transforms into a random coil. The water-holding capacity of collagen samples varies with the crosslinking method, as it depends on the number of available hydrogen bonds [45]. The samples lose 7–10% of the initial mass up to 135 °C, the process accompanied by a weak endothermic effect with a minimum between 71 and 75 °C (I.1–I.3). The process can be assigned to the elimination of weakly bound water from the sample structure (humidity and some hydrogen-bonded molecules). For sample I.4 with a higher mass loss, the peak of the endothermic effect is shifted toward a higher temperature, 84.8 °C. The second mass loss step between 135 and 400 °C is a complex process of structural water removal, partial denaturation of collagen by degradation of the polypeptide chain, and partial oxidation of the fragments as indicated by the multiple exothermic peaks on the DSC curves, with a visible maximum around 290–340 °C. The third mass loss, after 400 °C is generated by the complete oxidation of the residual carbonaceous mass but also by the HAp formation, densification, and –OH moiety elimination at the particle surface, the process accompanied by a strong exothermic effect with a maximum around 510–533 °C. The estimated HAp content for the samples Coll/HAp 3:1 is ~19–22%.

As for the samples Coll/HAp 3:1, the samples with Coll/HAp 1:1 lose 7–9% of the initial mass up to 135 °C, the process accompanied by a weak endothermic effect with a minimum between 75 and 80 °C, as shown in Table 4. The process can be assigned to the elimination of weakly bound water from the sample structure (humidity and some hydrogen-bonded molecules). As for the first series, the II.4 sample has the highest temperature for this endothermic peak, indicating a denser structure for genipin cross-linked samples. The small variation in the first mass loss step between samples from series I and II indicates that most of the water originates from humidity and not from collagen itself, as the larger proportion of collagen from series I does not seem to have an influence.

By contrast, the second mass loss step between 135 and 400 °C is smaller for series II, in concordance with its lower collagen content. This step is a complex process of structural water removal, partial denaturation of collagen by degradation of the polypeptide chain, and partial oxidation of the fragments as indicated by the asymmetric exothermic peaks on the DSC curves, with a visible maximum around 333–350 °C. The third mass loss, after 400 °C is generated by the complete oxidation of the residual carbonaceous mass but also by the HAp formation, densification, and –OH moiety elimination at the particle surface, the process is accompanied by a large and asymmetric exothermic effect with a maximum around 497–520 °C. The weaker intensity vs. series I for this effect indicates that the predominant source was the complete oxidation of the residual carbonaceous mass originating from collagen. The estimated HAp content for the samples Coll/HAp 1:1 is ~42–44%.

### 3.7. Mechanical Testing

**Figure 13:**
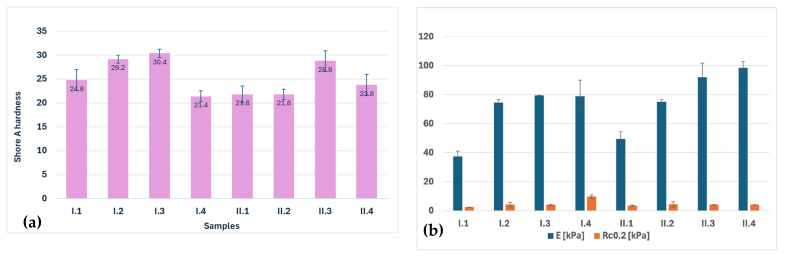
(**a**) Shore A hardness and (**b**) Young’s modulus and compression yield strength of composite scaffolds.

The mechanical properties of Coll/HAp composite materials are presented in Figure 13. As can be observed in Figure 13a, Shore A Hardness of the composite structures is situated between ~21 and 30, which is specific to a very soft and flexible material. These types of materials could be used as bone scaffolds in low-stress areas and the early stages of bone regeneration, as their compression yield strength is also not very high. Figure 13b illustrates the Young’s modulus and compression yield strength of the obtained scaffolds. Young’s modulus represents the stiffness of the materials, which indicates their resistance to deformation when subjected to an applied force. It is placed between 37.5 and 79 kPa for series I and 79.5 and 98.5 kPa for series II, the lowest values belonging to uncrosslinked samples. The behavior is characteristic of porous materials. First, a compression of the pores occurs, followed by their closure and the material’s densification. Increasing the hydroxyapatite content seems to have an influence on increasing the mechanical characteristics; it seems to slightly increase the stiffness and the compressive yield strength. A scaffold with a low Young’s modulus is more suitable for the regeneration of spongy bone, as it better mimics the mechanical environment and provides a more natural substrate for cells. A soft scaffold that has a low Young’s modulus can enhance cell adhesion, proliferation, and differentiation of osteoblasts and nutrient diffusion. Also, scaffolds characterized by a lower modulus enable the mechanical signals experienced by cells to mimic those of natural bone tissue. Considering that compression yield strength is not very high these scaffolds are more suitable to be used in early-stage bone healing, where the scaffold supports cellular infiltration and differentiation, allowing the scaffold to ultimately degrade as new bone forms. A solution to increase the mechanical properties could be the further mineralization of the scaffolds by immersion in SBF prior to implantation.

### 3.8. Antimicrobial Activity

**Figure 14:**
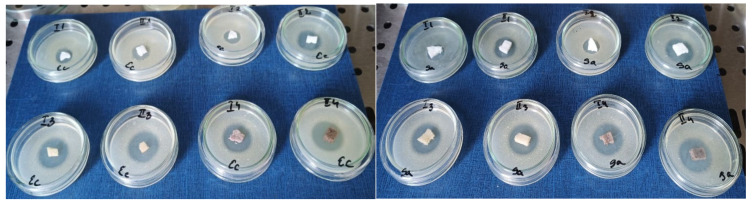
The antibacterial effect of Coll/HAp samples on *E. coli* (**left**) and *S. aureus* (**right**) strains.

Infection is one of the main risks associated with bone graft surgery. A 16-year study on a population subjected to bone grafting surgery revealed that the rate of infection was 3.05% [47]. The most common causative microorganism associated with graft infections is *S. aureus* [48]. In Figure 14, it can be observed that all samples possess antimicrobial activity.

The uncrosslinked samples (I.1 and II.2) show the lowest effect on both strains, although sample II.2, with a higher hydroxyapatite content, has a higher diameter of the inhibition zone (Table 5). The observation is confirmed by all samples from series II (with higher hydroxyapatite content) probably due to HAp influence such as pH alteration and ion release. HAp is able to slightly raise the local pH through the releasing of certain ions (calcium and phosphate), which generates a microenvironment less suitable for microorganism growth [49].

The crosslinked samples have high antimicrobial effects, the diameter of the inhibition zones being ≥20 mm. These results are due to the activity of crosslinking agents at the mechanisms of action and may be related to protein binding and membrane interactions (disruption of bacterial wall). Moreover, the antimicrobial activity seems to be more intense on the *S. aureus* strain, which is a great starting point considering that most graft infections are caused by this type of bacteria.

**Figure 15:**
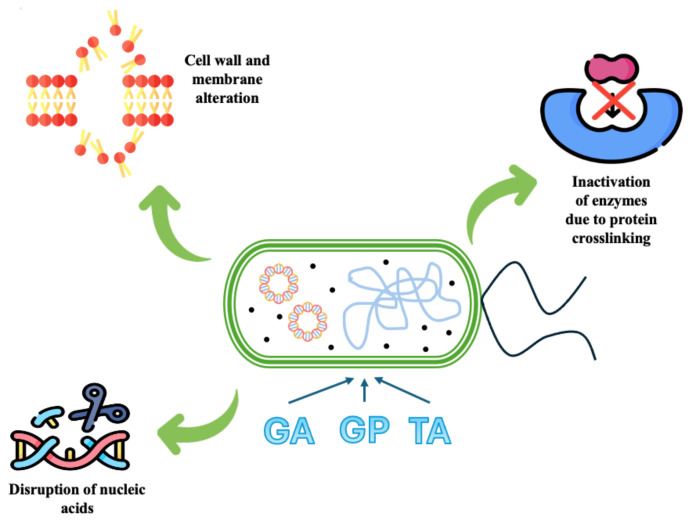
The mechanism of antimicrobial activity.

The antimicrobial activity of the obtained composite materials is first of all attributed to the ability of tannic acid, genipin, and glutaraldehyde to directly bind to the bacterial peptidoglycan layer, leading to structural modifications [50,51]. Additionally, these compounds promote protein crosslinking, causing the inactivation of enzymes that are essential for survival [31,52]. Figure 15 shows the mechanism of antimicrobial activity. In the case of phenolic compounds, they damage bacterial processes by being involved in DNA synthesis and regulation. Their structure, containing hydroxyl groups and an aromatic ring, allows polyphenols to interact with amino or carboxylic groups of proteins. By modifying cell membrane permeability, polyphenols can determine the leakage of intracellular components, including DNA. Glutaraldehyde also interacts with nucleic acids, such as DNA and RNA, via its aldehyde groups, compromising nucleic acid integrity and impairing critical enzymes and cellular functions [53]. Phenolic compounds also exhibit antibacterial effects by inhibiting enzymatic activity. This inhibition appears from protein–phenolic interactions, which occur through covalent or noncovalent bonds and depend on the structural properties of the proteins, including hydrophobicity, molecular weight, conformational arrangement, amino acid composition, and sequence [54,55]. The reactions that take place between polyphenols and sulfhydryl (SH) groups of proteins, as well as non-specific interactions, represent another mode of enzyme inhibition. The extent of inhibition is influenced by the number of hydroxyl groups, with highly oxidized polyphenols exhibiting greater toxicity toward microorganisms [56].

## 4. Conclusions

The present study focused on the development of collagen and hydroxyapatite-based composite materials, which can be used for bone tissue engineering and further in cancer treatment after the incorporation of anticancer agents. The samples were crosslinked with three different agents and obtained with a freeze-drying method. Results showed promising results; the morphology of the scaffolds showed a high porosity with interconnected pores suitable for osteoblast infiltration and proliferation. The hydroxyapatite phase formation that was obtained in situ starting from Ca and P precursors was confirmed and showed a homogeneous distribution. Moreover, the scaffolds (especially those cross-linked with the two natural agents, tannic acid and genipin) presented a very good antibacterial activity against *E. coli* and *S. aureus* bacterial strains, indicating a great potential as antimicrobial composites. There is still room for the improvement of these scaffolds, especially in terms of mechanical properties, if they are intended to be used in areas with higher mechanical stresses. We concluded that the best material composites are those crosslinked and with higher amounts of hydroxyapatite (series II), and they will be further analyzed to determine their cytocompatibility. The most promising composite materials will be loaded with a mixture of drugs and applied in the treatment of bone-related infections or cancers.

## Figures and Tables

**Table 1:** Codification and composition of Coll/HAp composite materials.

Sample Code	Coll/HAp 3:1 Mineralized Gel	Coll/HAp 1:1 Mineralized Gel	Glutaraldehyde 0.2%	Tannic Acid 1%	Genipin 1.5%
I.1	9 mL	-	-	-	-
I.2	9 mL	-	1 mL	-	-
I.3	9 mL	-	-	1 mL	-
I.4	9 mL	-	-	-	1 mL
II.1	-	9 mL	-	-	-
II.2	-	9 mL	1 mL	-	-
II.3	-	9 mL	-	1 mL	-
II.4	-	9 mL	-	-	1 mL

**Table 2:** Elemental composition of sample II.4.

Element	Weight %	Atomic %	Error %
C K	22.63	33.83	12.54
O K	43.26	48.55	11.18
Na K	3.03	2.37	14.86
P K	9.08	5.26	5.4
Cl K	2.17	1.1	7.23
Ca K	19.83	8.88	2

**Table 3:** The main data obtained from the thermal analysis of series I—Coll/HAp 1:3 samples.

Sample	Mass Loss % RT-135 °C	Endo I °C	Mass Loss % 135–400 °C	Mass Loss % 400–900 °C	Residual Mass %	HAp % (rez 92%)
I.1	8.70	72.1	38.73	33.69	18.88	20.52
I.2	7.82	71.4	39.56	33.16	19.41	21.10
I.3	6.93	74.7	38.78	34.18	20.09	21.84
I.4	9.82	84.8	36.60	35.63	17.94	19.50

**Table 4:** The main data obtained from the thermal analysis of series II—Coll/HAp 1:1 samples.

Sample	Mass Loss % RT-135 °C	Endo I °C	Mass Loss % 135–400 °C	Mass Loss % 400–900 °C	Residual Mass %	HAp % (rez 92%)
II.1	6.91	79.0	32.89	19.79	40.41	43.92
II.2	8.63	78.6	33.24	18.20	39.92	43.39
II.3	7.65	75.1	33.77	18.85	39.72	43.17
II.4	7.92	80.5	33.37	19.40	39.27	42.68

**Table 5:** The evaluation of the results according to the diameters of the inhibition zones: 10–14 mm, weak activity—marked “+”; 15–19 mm, moderate activity—marked “++”; ≥20 mm, very good activity—marked “+++”.

Sample	Result	
	*Escherichia coli* ATCC 10536	*Staphylococcus aureus* ATCC 6538
I.1	+	+
II.1	++	+++
I.2	++	++
II.2	+++	+++
I.3	+++	+++
II.3	+++	+++
I.4	+++	+++
II.4	+++	+++

## Data Availability

The original contributions presented in the study are included in the article, further inquiries can be directed to the corresponding author.

## Appendix A

**Table A1:** The water uptake of series I at intermediate times.

Time	I.1	SD	I.2	SD	I.3	SD	I.4	SD
1 h	32.3854	1.8455	27.3102	1.3949	33.9259	2.8628	22.6202	0.9752
2 h	36.5731	1.6855	28.1749	1.316	34.1771	1.6804	25.4603	3.2604
4 h	36.0146	2.8666	28.1265	1.8716	35.4709	1.9908	25.2210	3.2299
24 h	34.5932	1.5981	27.686	2.6646	35.8255	0.7345	25.6504	3.1545
48 h	36.8161	4.6903	30.9512	1.6697	37.9903	0.5637	28.3459	0.8751

**Table A2:** The water uptake of series II at intermediate times.

Time	II.1	SD	II.2	SD	II.3	SD	II.4	SD
1 h	23.8703	3.4107	22.9792	3.6308	25.3585	1.2655	28.9006	1.3819
2 h	23.0132	4.6984	25.0582	3.0948	24.2272	1.3135	30.8481	4.7348
4 h	24.7849	1.6964	25.7003	2.5981	22.6525	1.6317	29.3223	1.0392
24 h	25.582	2.6111	23.9612	3.8225	23.1907	0.8352	30.2634	1.5926
48 h	22.6094	1.6101	26.8037	1.3471	23.5970	0.3977	29.9090	1.5276
